# Cultivation of *E. coli *carrying a plasmid-based Measles vaccine construct (4.2 kbp pcDNA3F) employing medium optimisation and pH-temperature induction techniques

**DOI:** 10.1186/1475-2859-10-16

**Published:** 2011-03-05

**Authors:** Clarence M Ongkudon, Raelene Pickering, Diane Webster, Michael K Danquah

**Affiliations:** 1 Bio Engineering Laboratory, Department of Chemical Engineering, Monash University, Clayton campus, Wellington road, Victoria 3800, Australia; 2 School of Biological Sciences, Faculty of Science, Monash University, Clayton campus, Wellington road, Victoria 3800, Australia

## Abstract

**Background:**

Plasmid-based measles vaccines offer great promises over the conventional fertilised egg method such as ease of manufacture and mimic wild-type intracellular antigen expression. The increasing number of clinical trials on plasmid-based measles vaccines has triggered the need to make more in less time.

**Results:**

In this work, we investigated the process variables necessary to improve the volumetric and specific yields of a model plasmid-based measles vaccine (pcDNA3F) harboured in *E. coli *DH5*α*. Results from growth medium optimisation in 500 mL shake flasks by response surface methodology (RSM) generated a maximum volumetric yield of 13.65 mg/L which was 1.75 folds higher than that of the base medium. A controlled fed-batch fermentation employing strategic glycerol feeding and optimised growth conditions resulted in a remarkable pcDNA3F volumetric yield of 110 mg/L and a specific yield of 14 mg/g. In addition, growth pH modification and temperature fluctuation between 35 and 45°C were successfully employed to improve plasmid production.

**Conclusion:**

Production of a high copy number plasmid DNA containing a foreign gene of interest is often hampered by the low plasmid volumetric yield which results from the over expression of foreign proteins and metabolic repressors. In this work, a simple bioprocess framework was employed and successfully improved the production of pcDNA3F.

## Background

Plasmid DNA (pDNA) vaccine is a third generation of vaccine technology which offers an attractive new alternative to conventional immunisation techniques. In human trials, pDNA has been shown to induce protective immunity similar to that of natural infection for not only measles, but across a broad range of virus families [1]. From a production stand point, the lyophilised form of the current vaccine lacks thermal stability, requiring an uninterrupted cold chain for maximum efficacy [2]. The enhanced thermal stability of plasmid DNA at room temperature and above offers a great promise for the treatment of measles and other diseases in tropical and economically disadvantaged areas [3]. General steps involve in the production of plasmid vaccines are similar to that of protein production that include fermentation, primary isolation and purification [4]. It is presumed that the mechanisms that contribute to yield improvement are reduced metabolic burden during plasmid synthesis; reduced plasmid mediated protein production and altered DNA compaction during plasmid induction [5]. Various bioprocess engineering approaches that can be employed to alter the growth of *E. coli *hence gene expression have extensively been discussed by Razali *et al. *[6] that include temperature shift techniques, feeding strategies, timing of induction and plasmid stabilisation.

It is important to note that like chromosomal DNA, plasmid DNA is made up of sugar-phosphate backbone and nitrogen base nucleotides (ATGC). Carbon, phosphorus and nitrogen are the main ingredients in DNA biopolymers unlike proteins. Also, in the central dogma of molecular biology, only replication is required for DNA synthesis whilst transcription and translation are added mechanisms for protein synthesis. Thus, cultivation medium for DNA production in *E. coli *is substantially different from protein production [7]. In our previous work [8], we have developed an economically viable semi-complex stoichiometric medium for pUC based plasmid production. In this work, we used the same media and optimised it specifically for plasmid-based Measles vaccine (pcDNA3F) production. Growth temperature up-shifts may be employed to induce plasmid replication and reduce contaminating RNAs and gDNAs by down regulating the growth rate of the cells [3,4]. Several researchers have attempted multiple temperature induction schemes that included constant, gradual and fluctuating temperature shifts specific to a product expression [9-11]. The duration at which the cells are exposed to a certain temperature shift is known to affect the maximum recombinant protein production [12,13]. The use of fluctuating temperatures to achieve selective gene expression has also been reported in other biological systems [14,15]. It is further suggested that a cooling step may be required to drive cells to maximum potential plasmid copy number which may not be possible at higher temperatures [16].

Production of extended cell density and growth-related pDNA yield can be achieved in a fed-batch culture by expanding the cultivation time under the controlled provision of substrates (e.g. glucose, phosphate and oxygen) conducive to pDNA formation and cell growth [3]. A good fed-batch fermentation practice is based on the supplementation of a substrate at a rate such that it is completely consumed. The fermentation commences with a batch mode under non-limiting conditions and an optimum cell growth rate. When one or more substrates are exhausted, the batch mode is then switched to fed-batch mode [17]. Several methods have been suggested to synchronize substrate feed and demand including an open-loop control scheme where the feeding rate is controlled based on previously established data or fixed model [5,18-20] and an indirect control of substrate feed based on on-line physiological parameters such as pH, dissolved O_2 _and cell concentration [3,17]. A rational equation based on the biomass yield coefficient Y_X/S _(g DCW/g substrate) may be employed for a better control of the substrate feeding. In this article, we present bioprocess methods for the improvement of volumetric and specific yields of a model plasmid-based measles vaccine production. It involves medium optimisation, growth pH alteration, varied temperature shifting, fed-batch cultivation and glycerol exponential feeding strategy specific to pcDNA3F-harbouring *E. coli *DH5α cells. pcDNA3F was derived from plasmid pcDNA3 (Invitrogen) and fusion protein gene (F) of measles virus. This work was motivated by the fact that under a pre-defined culture condition, pcDNA3F yields were lower than that of pcDNA3. Consequently, methodologies and results from this study may be used to favour the subsequent development of commercial scale production of pcDNA3F.

## Results and discussions

### Medium optimisation

The basic composition of pUC-based plasmid DNA semi-defined medium (PDM) as depicted in Table 1 was reported by [7] which was developed from a stoichiometric analysis. Prior to the medium optimisation, a preliminary medium screening based on Plackett Burman design [21,22] was carried out to select three most significant medium components in PDM and the results showed that tryptone, glucose and disodium hydrogen orthophosphate (Na_2_HPO_4) _significantly affected pcDNA3F volumetric yield (data not shown). Following the medium screening, a series of 16 central composite design (CCD) [23] experiments as shown in Table 2 were conducted to optimise tryptone, glucose and disodium hydrogen orthophosphate (Na_2_HPO_4_) contents in PDM. The concentrations of tryptone, glucose, and Na_2_HPO_4 _in each experiment were varied from 0.5 g/L (coded value = -2) to 18.5 g/L (coded value = +2). The central value of 10 g/L (coded value = 0) was chosen as the average concentration of tryptone, glucose, and Na_2_HPO_4 _in PDM. The parameters' coefficients obtained from the ANOVA (Table 3) were used to construct the second-order polynomial model which explained the correlation of each nutrient and their second-order interactions with the plasmid volumetric yield. Thus, pcDNA3F volumetric yields were predicted by the following equation.(1)

where *a_0, _a_1, _a_2, _a_3, _a_4, _a_5, _a_6, _a_7, _a_8, _a_9 _*are parameters' coefficients as shown in Table 3, *x_1 _*is the coded value of tryptone concentration, *x_2 _*is the coded value of glucose concentration, *x_3 _*is the coded value of Na_2_HPO_4 _concentration and *y *is the pcDNA3F volumetric yield (mg/L). The quadratic model in equation (1) involves nine terms consisting of three linear terms, three quadratic terms, and three two-factor interactions. All terms were included in the model to give the best fit of the experimental data. Generally, the equation shows a good agreement between predicted and observed experimental data (Table 2). The lack of fit of the model was checked by the determination coefficient (*R*^2^) which is the ratio of *SS *(regression) to *SS *(total). *R*^2 ^is a measure of the amount of reduction in the variability of *y *obtained by using the parameters' coefficients in the model. The value of the determination coefficient (*R*^2 ^= 0.95) indicates that 95% of the data are well matched by equation (1). The significance of each coefficient was determined by student's *t*-test, *F *and *P *values, which are listed in Table 3.

The *F *values show how larger the mean square of the coefficients compared to mean square of the residuals (errors). The significance of *F *value or sometimes referred to as *P *value is the probability to get larger *F *value by chance alone. The larger the magnitude of the *t *and *F *values and the smaller the *P *value, the more significant the corresponding coefficient is. As a rule of thumb, coefficients with *P *< 0.05 are considered significant. In general, the linear (*x_1_, x_2_, x_3_*) and quadratic (*x_1_^2^, x_2_^2^, x_3_^2^*) interactions between the coefficients and plasmid yield are significant thus contribute to the adequacy of the regressed model. The magnitudes of the linear and quadratic terms are evenly large which show that they all contributed significantly to the plasmid volumetric yield. The negative values of the quadratic terms also indicate that there are maximum values for each nutrient's concentration. Based on the corresponding *t*, *F *and *P *values, Na_2_HPO_4 _seems to have the most profound impact on pcDNA3F volumetric yield. This is true since phosphate forms a major component in the plasmid structure. Meanwhile, the two-way interaction between tryptone (*x_1_*) and Na_2_HPO_4 _(*x_3_*) yields the lowest *F *value. This indicates that both nutrients have a distinct role in plasmid synthesis possibly due to lack of phosphate in tryptone. Glucose (*x_2_*) and tryptone (*x_1_*) also have insignificant interaction possibly due to lack of carbon source in tryptone while glucose (*x_2_*) and Na_2_HPO_4 _(*x_3_*) generally have a high degree interaction possibly because glucose and Na_2_HPO_4 _are the main components in plasmid backbone.

RSM was carried out to predict the highest point of plasmid yield as well as optimum values of tryptone, glucose and Na_2_HPO_4 _concentration using equation (1). The optimum values of the parameters in coded values are *x*_1 _= 0.12, *x*_2 _= -0.69, and *x*_3 _= 1.13 with the corresponding response y = 12.59 mg/L. This translates to real values of the test variables which are given in Table 1. Actual experiments using the optimised medium were carried out and the results showed that the optimised medium for pcDNA3F cultivation resulted in an improved volumetric yield of 13.65 ± 0.10 mg/L which is 1.75 folds higher than the non-optimised medium (7.80 ± 0.2 mg/L). In summary, the utilisation of experimental design and RSM on three important factors of the plasmid DNA medium resulted in a markedly improved plasmid pcDNA3F yield.

To evaluate the performance of the optimised medium against commercial medium, *E. coli *carrying pcDNA3F was cultured in different commercially available growth media. Parameters such as plasmid volumetric and specific yields as well as production rates were compared and the results are shown in Table 4. Although the results are mixed, but it can immediately be seen that the optimised medium (PDM-pcDNA3F) has significant advantages over other medium in terms of highest plasmid production rate and specific yield.

**Table 1:** Composition of basic (PDM) and optimised (PDM-pcDNA3F) medium for DH5α-pcDNA3F cultivation.

Nutrient	PDM (g/L)	PDM-pcDNA3F (g/L)
yeast extract	4.41	4.41
tryptone	7.93	10.60
glucose	10.00	6.50
Na_2_HPO_4_	12.80	15.70
K_2_HPO_4_	3.00	3.00
NH_4_Cl	0.50	0.50
MgSO_4_	0.24	0.24

**Table 2:** Central composite design for the optimization of tryptone, glucose and disodium hydrogen orthophosphate concentrations.

	Coded Values*			Real Values*			*y *= pDNA (mg/L)	
				g/L	g/L	g/L		
**Run no**.	*x_1_*	*x_2_*	*x_3_*	*X_1_*	*X_2_*	*X_3_*	Actual	Predicted
1	-1	-1	-1	5	5	5	6.99	6.75
2	-1	-1	1	5	5	15	11.7	10.98
3	-1	1	-1	5	15	5	6.03	6.06
4	-1	1	1	5	15	15	7.95	7.74
5	1	-1	-1	15	5	5	7.65	7.2
6	1	-1	1	15	5	15	12.03	11.31
7	1	1	-1	15	15	5	8.43	8.46
8	1	1	1	15	15	15	10.44	10.02
9	-1.7	0	0	1.5	10	10	6.27	6.6
10	1.7	0	0	18.5	10	10	8.34	8.94
11	0	-1.7	0	10	1.5	10	9.06	9.96
12	0	1.7	0	10	18.5	10	8.25	8.28
13	0	0	-1.7	10	10	1.5	6.66	6.69
14	0	0	1.7	10	10	18.5	10.71	11.61
15	0	0	0	10	10	10	11.79	11.55
16	0	0	0	10	10	10	11.43	11.55

* *x_1_, x_2 _*and *x_3 _*are coded values of tryptone, glucose and disodium hydrogen orthophosphate concentrations respectively whilst *X_1_, X_2 _*and *X_3 _*correspond to real values of tryptone, glucose and disodium hydrogen orthophosphate concentrations respectively.

**Table 3:** ANOVA.

Parameter*	Coefficients, *a_n_*		*SS*	*t*-Stat	*MS*	*F*	*P*
Intercept	*a_0 = _*	11.55		20.99			0.00
*x_1_*	*a_1 =_*	0.69	6.41	3.25	6.41	10.54	0.02
*x_1. _x_1_*	*a_2 =_*	-1.32	16.35	-5.19	16.35	26.88	0.00
*x_2_*	*a_3 =_*	-0.51	3.48	-2.38	3.48	5.68	0.05
*x_2. _x_2_*	*a_4 =_*	-0.84	6.78	-3.34	6.78	11.15	0.02
*x_3_*	*a_5 =_*	1.44	28.75	6.88	28.75	47.28	0.00
*x_3. _x_3_*	*a_6 =_*	-0.84	6.61	-3.30	6.61	10.87	0.02
*x_1. _x_2_*	*a_7 =_*	0.48	1.90	1.77	1.90	3.13	0.13
*x_1. _x_3_*	*a_8 =_*	-0.03	0.01	-0.11	0.01	0.01	0.92
*x_2. _x_3_*	*a_9 =_*	-0.66	3.33	-2.34	3.33	5.47	0.06
	residual		6.08		6.08		
	*R*^2^		94.60				
	Adjusted *R*		86.78				

*x_1 _is the coded value of tryptone concentration, *x_2 _*is the coded value of glucose concentration and *x_3 _*is the coded value of Na_2_HPO_4 _concentration.

**Table 4:** Analysis of plasmid production and cell growth in different commercially available media and PDM-pcDNA3F.

Medium	pcDNA3F volumetric yield*	pcDNA3F specific yield*	pcDNA3F production rate*	Final cell density*	Cell growth rate*
	(mg/L)	(mg/g)	(mg/L/h)	(g/L)	(h^-1^)
Terrific Broth	8.40	4.31	0.56	1.95	0.85
Luria Broth	5.80	7.94	0.39	0.73	0.31
2xYT	6.42	3.82	0.43	1.68	0.77
PDM	7.80	3.57	0.52	2.18	0.89
PDM-pcDNA3F	13.65	9.10	0.91	1.50	0.75

*Cells were cultured in 250 ml of medium containing 100 μg/mL ampicillin using 500 mL shake flasks at 200 rpm, 37°C and halted after 15 h. Each value represents an average of triplicates. Standard deviations for all data are 1 - 5% of the values shown.

### Effect of growth conditions on cell growth and pcDNA3F production

**Figure 1:**
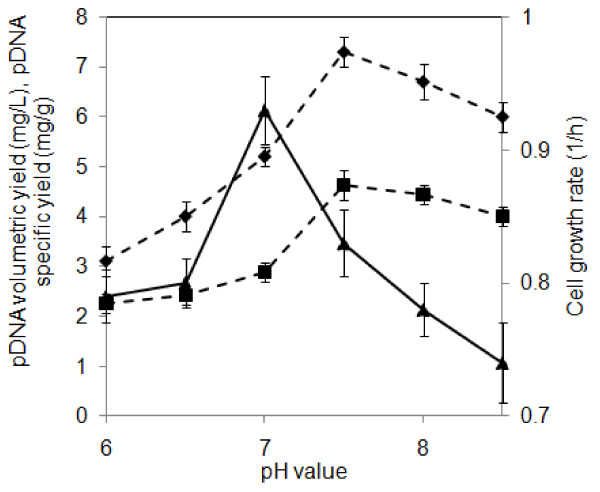
**Effect of pH on cell growth rate and plasmid yield**. Cells were cultured in 250 ml of PDM medium using 500 mL shake flasks at 200 rpm, 37°C and halted after 15 h. Each data point represents an average of triplicates. Rhombus: pcDNA3F volumetric yield; square: pcDNA3F specific yield; triangle: cell growth rate.

*E. coli *has traditionally been grown at pH~7. In plasmid DNA production, it is conjectured that a moderate pH deviation might have a positive effect on plasmid yield. Thus, pcDNA3F volumetric and specific yields were assessed at different initial pH ranging from 6 to 8.5. The result as shown in Figure 1 demonstrates that reduced cell growth rate does not necessarily followed by an increase in pcDNA3F yield. Generally at lower pH, cell growth and pcDNA3F production were suppressed whilst at higher pH; pcDNA3F yield was improved with decreased cell growth. At pH 6 - 7, pcDNA3F yield was dependent on biomass yield; thus plasmid yield increased as maximum cell growth rate increased. At pH 7 - 7.5, a non-growth dependent or inverse correlation profile was displayed. A maximum cell growth rate of ~ 0.9 h^-1 ^was obtained at pH 7 where pcDNA3F volumetric and specific yields were ~ 5 mg/L and ~ 3 mg/g respectively. Optimum pcDNA3F yield of ~ 7 mg/L (4 - 5 mg/g) was achieved at pH 7.5 when the cell growth rate was only 0.8 h^-1^. From a molecular perspective, pH gradient across a cell membrane is theoretically related to the generation of proton motive force (PMF). Subsequently, PMF is likened to the production of ATP for use in different cellular metabolic pathways including DNA replication. It is presumed that when the extracellular pH condition deviates from neutral to acidic condition, PMF hence ATP drastically drop and suppress both cell growth and plasmid DNA synthesis. This profile is indicated in the pH range of 6 to 7 of Figure 1. This can be explained by the impairment of ATP production at acidic condition as a result of fueling patways and enzyme inhibition as described by [24,25]. Under a low pH stress, ATPases are activated to compensate for pH variations in the cytoplasmic region by proton intrusion into the periplasm with the concomitant consumption of ATPs [25,26]. However, it is speculated that a small pH deviation to the alkaline region is needed to generate PMF which preferentially improves plasmid yield over cell growth. In other words, optimum synchronization of cell growth, plasmid synthesis and PMF is achievable in a slightly alkaline condition. These results of effect of pH on plasmid production however, are rather contradictory to studies reported by [27]. In their studies, plasmid pEGFP-N1 was cultivated at initial pH values ranging from 6.5 to 8.5 and the highest plasmid specific yield was obtained at a slightly acidic condition at pH 6.5.

**Figure 2:**
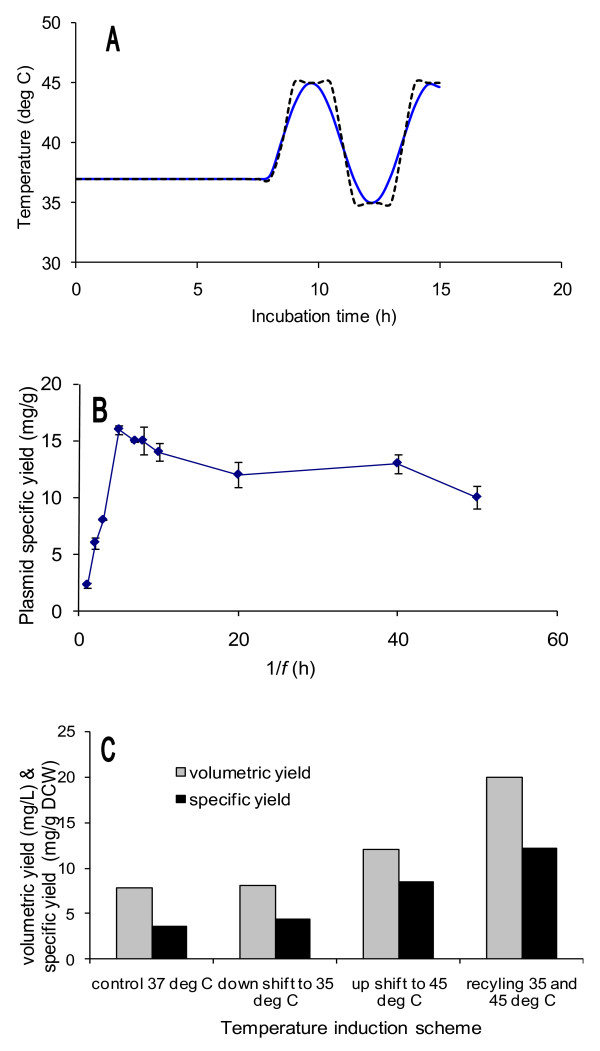
**Effect of different growth temperature induction schemes on plasmid production**. Temperature inductions were performed at 8 h post inoculation. Cells were cultured in 250 ml PDM medium using 500 mL shake flasks at 200 rpm, initial temperature of 37°C and halted after 15 h. Each data point represents an average of five replicates (n = 5). (A) Profiles of an actual (dashed line) and a model (solid line) growth temperature fluctuations. (B) Effect of frequency of temperature fluctuation (1/*f*) on plasmid specific yield. (C) Analysis of plasmid volumetric and specific yields from different temperature induction schemes.

A unique temperature fluctuation (Figure 2A) was conducted to increase plasmid productivity theoretically by inactivating the synthesis of repressor at higher temperatures and periodically maintaining plasmid stability at lower temperatures. The solid line in Figure 2A represents equation (2) where *T*(*t*) is temperature at time *t*, *T*(0) is based temperature, Δ*T *is temperature deviation, *f *is frequency of temperature fluctuation, and *ϕ *is phase at the start of the temperature shift.(2)

The duration of temperature shift exposure as indicated by the reciprocal of frequency *f *in equation (2) shows an optimum value of 1/*f *= 5 h corresponding to an optimum plasmid specific yield of ~ 15 mg/g DCW (Figure 2B). In addition to that, the use of controlled temperature fluctuation shows great advantages over single step temperature shifts in terms of maximum plasmid volumetric and specific yields (Figure 2C). Cells exposed to a high temperature regime exhibit diminished growth which can be explained by the over expression of foreign proteins and metabolic repressors [28,29]. An increase in growth temperature results in cloned gene expression as a result of promoter activation which in turn triggers expression of growth inhibitors and metabolic repressors [29]. Plasmid DNA being a temperature stable molecule continues to replicate under this condition. However, prolonged exposure of conditions that derepress the promoter can lead to a decreased ratio of plasmid-harbouring cells[9]. High temperature is also associated with low oxygen solubility and decreased TCA activity hence higher acetate production[10] as well as inhibition of cell wall synthesis leading to filamentation and reduced cell growth [30]. It is thought that returning the culture to a lower temperature regime just before the accumulation of plasmid segregants favours high level of plasmid-harbouring cells for an extended period of time hence higher plasmid volumetric and specific yields are achieved.

### Fed-batch fermentation

**Figure 3:**
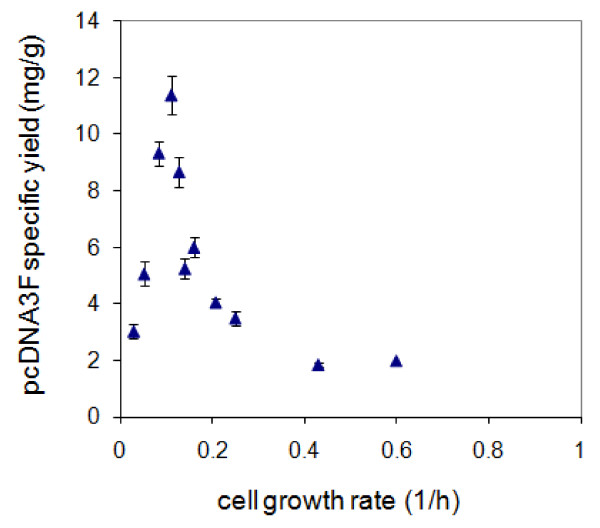
**Effect of cell growth rate of *E. coli *DH5α on pcDNA3F specific yield**. Data were obtained from batch fermentations at different initial glucose concentrations. Each data point represents an average of triplicates.

Prior to performing the fed-batch fermentation, the effect of *E. coli *cell growth rate on pcDNA3F specific yield (Figure 3) was studied by growing the culture in batch mode in PDM medium containing different initial concentrations of glucose. Samples were collected every 30 min during the batch fermentations and analysed for cell concentration and pcDNA3F volumetric yield. Glucose was found to affect the cell growth rate (μ) and the final biomass concentration which in turn resulted in different levels of pcDNA3F specific yield as depicted in Figure 3. A maximum pcDNA3F specific yield of 11 mg/g was achieved at a considerably low cell growth rate of 0.1 h^-1^. The strong dependency of plasmid specific yield on cell growth rate was evident since a drastic drop in specific yield was observed even at a slight deviation of the cell growth rate. We speculate that the molecular mechanism contributing to this phenomenon is associated with the synchronization of cell growth and plasmid replication. pcDNA3F plasmid is constructed based on pUC plasmid which has high copy number due to the removal of protein repressor (Rop/Rom) gene from its genetic sequence. To reach at the maximum plasmid copy number, it has to undergo multiple cycles of RNA transcription, DNA pairing and ligation in a single *E. coli *cell. Therefore, cell replication has to be slowed down to give time for the plasmid to complete its continuous extra chromosomal replication.

**Figure 4:**
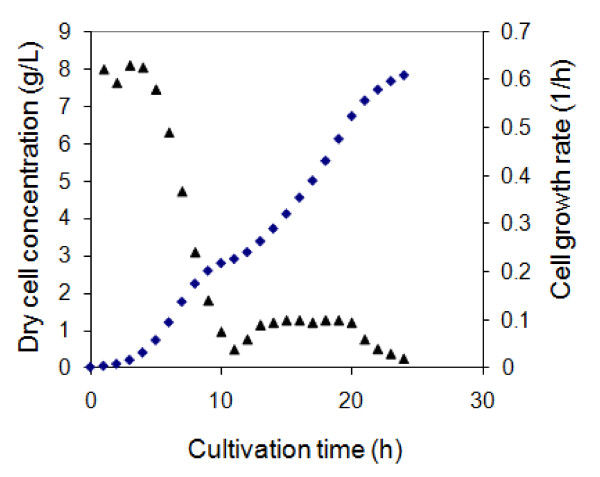
**Dry cell concentration and cell growth rate profiles during fed-batch fermentation of *E. coli *DH5α-pcDNA3F**. Glycerol feeding was initiated at 10 h post culture and temperature upshift (37°C to 45°C) was performed at 20 h post inoculation. Rhombus: dry cell concentration; triangle: cell growth rate.

Batch fermentation was used to initiate a culture under a non limiting environment. This was to accumulate the biomass density within a short period of time thus reducing the initial fermentation cost. In batch fermentation, the cells were cultivated in PDM-pcDNA3F medium at a cell growth rate of 0.6 h^-1 ^which was equivalent to a biomass doubling time, t_d _of 1.1 h (Figure 4). As the fermentation progressed, there was a downward trend of cell growth rate which was mainly due to a nutrient depletion. The decreased cell growth rate and increased biomass density both contributed to increase in plasmid volumetric and specific yields. When the carbon source was fully consumed, no significant increase in biomass density was observed. Fed batch process was then initiated under the controlled provision of glycerol into the fermentor.

During the fed-batch phase, the amount of substrate (glycerol) fed into the bioreactor was controlled via an automated peristaltic pump according to equation (3) where *F_t _*is the substrate feeding rate at *t *h after the fed-batch mode is initiated, *F_i _*is the initial substrate feeding rate, *α *is the specific exponential feeding rate, and *t *is the cultivation time during the fed-batch phase. According to equation (3), *F_t _*can be optimised by fine-tuning the values of *F_i _*and *α*. The value of *F_i _*can be approximated by integration of equation (4) where *∫S_t _*is the cumulative amount of substrate consumed in the batch phase and *S_i _*is the initial substrate consumption rate in the batch phase.(3)(4)(5)

Therefore,(6)

**Figure 5:**
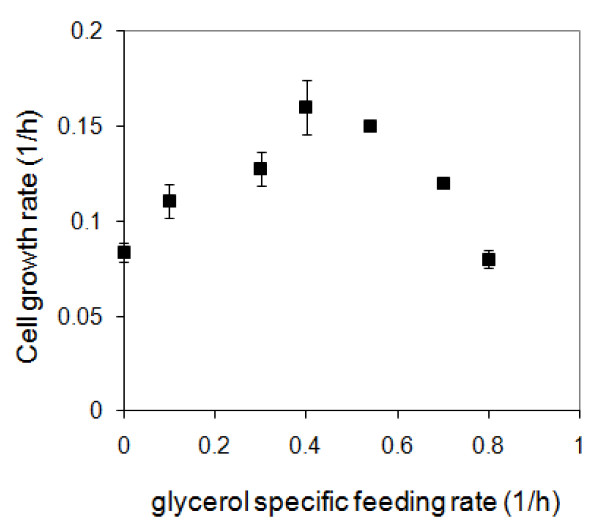
**Cell growth rates of *E. coli *DH5α carrying pcDNA3F at different glycerol exponential feeding rates, α**. Data were obtained from fed-batch fermentations at different glycerol feeding rates. Each data point represents an average of triplicates.

A series of experiments was conducted at different *α *values until the highest point of plasmid specific yield was obtained. Samples were collected every 30 min during the fed-batch phases at different *α *values and analysed for cell growth rate and the results are shown in Figure 5.

Glycerol exponential feeding strategy greatly affects the cells growth by maintaining the cell growth rate below 0.2 h^-1 ^with improved plasmid specific yields. Although a low cell growth rate is beneficial for plasmid replication, this may not hold true at *α *> 0.4. This is due to the significantly large amount of glycerol that needs to be pumped into the bioreactor every hour at *α *> 0.4. As the cultivation time *(t) *and exponential feeding rate (*α*) increase, the dilution rate (*D*) becomes extremely large and impractical. In this case, the optimum *α *value is ~0.1 h^-1 ^which is equivalent to a pcDNA3F specific yield of ~12 mg/g.

**Figure 6:**
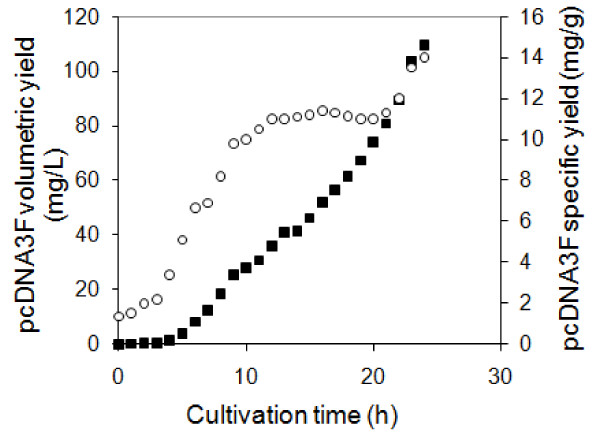
**pcDNA3F volumetric yield and specific yield profiles during fed-batch fermentation of *E. coli *DH5α-pcDNA3F**. Glycerol feeding was initiated at 10 h post culture and temperature upshift (37°C to 45°C) was performed at 20 h post inoculation. Square: volumetric yield; rhombus: specific yield.

In this work, the initial glycerol feeding rate was successfully estimated by using an exponential feeding rate equation and assuming that the carbon consumption rate equalled the cell growth rate (*μ*) in the batch phase. Therefore, the final carbon consumption rate in the batch phase was used as the initial glycerol feeding rate in the fed-batch phase. Using the calculated initial glycerol feeding rate and increasing it exponentially, the biomass proliferated at a reduced cell growth rate of 0.1 h^-1 ^(t_d _= 6.9 h) (Figure 4) conducive to pcDNA3F propagation. By maintaining this low cell growth rate throughout the fed-batch phase, a considerably high plasmid specific yield of about 12 mg/g was obtained (Figure 6). At 20 h post culture, the growth temperature was shifted from 37°C to 45°C. The final plasmid pcDNA3F yields showed a profound improvement compared to that was normally achieved in batch fermentations.

**Figure 7:**
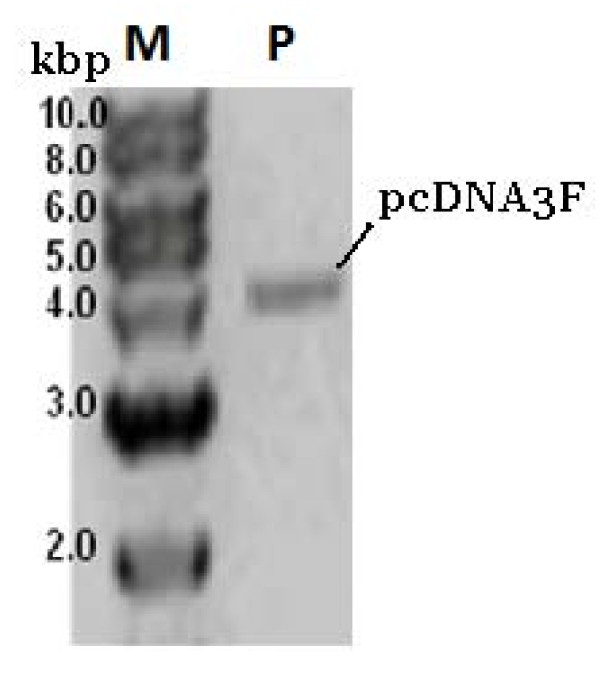
**Ethidium bromide agarose gel electrophoresis of pDNA samples from fed batch fermentation of DH5α-pcDNA3F**. Loading volume = 1 μL. Analysis was performed using 1% agarose in TAE × 1 buffer, 3 μg/mL ethidium bromide at 65 V for 90 min. Lane M is 1 kb DNA ladder and lane P represents 55.0 mg/L of purified pcDNA3F.

It has been reported elsewhere that the plasmid DNA medium can provide as high as 12 g/L dry cell concentration in a fed-batch system using pUC based plasmid system [7]. We speculated that the fairly low dry cell density obtained in this work using the same base plasmid was due to over expression of foreign gene (MV-F). At the start of the fed-batch mode, cells density was around 3 g/L. Further increase in cell density was observed at 12 h onwards before reaching 6 g/L at 19 h which correlated to a cell growth rate of 0.1 h^-1 ^and a biomass doubling time of 7 h. The fermentation was halted at 24 h (early stationary phase) which was equivalent to a final cell density of ~ 8 g/L. Again the harvest time was chosen as the optimum combination between cell growth and plasmid yield. At this point, one or more nutrients became limiting since only carbon substrate was provided in the fed-batch mode. DNA gel electrophoresis of purified pcDNA3F (Figure 7) showed pure supercoiled plasmid DNA with no nicked plasmid DNA isoforms.

Fed-batch cultivation with glycerol feeding commenced after an initial glucose carbon source in the initial batch fermentation phase has starved. Hence, the fed-batch mode occurred at a fairly high cell concentration in the late exponential phase. The inconsistency in the source of carbon for the cells during the batch and fed-batch contexts could result in the mismatched pattern in the somewhat initial low growth phase. However, glucose was not used in the fed-batch case as the interest here was to boost plasmid replication and not cell growth. Glycerol was used to tweak cell metabolism to support plasmid replication as the kinetics of glycerol transportation and uptake is slower than glycolysis.

## Conclusions

It has been observed that the inclusion of fusion protein gene (F) of measles virus into base plasmid pcDNA3 resulted in reduced plasmid volumetric yields during fermentation compared to naked pcDNA3. This was possibly due to extra factors introduced by the F gene into the culture which interfered with normal plasmid production and *E. coli *cell growth. Higher plasmid yields are often the results of higher plasmid stability and preferential plasmid synthesis over other biomolecules syntheses. Bioreactor and medium designs are known to have significant effects on plasmid yields. One of the unifying criteria for high purity and high yield plasmid cultivation is the use of defined or semi-defined medium that allows the cells to grow at a reduced cell growth rate whilst preferentially boosting plasmid replication rate. At a molecular level, the mechanisms that contribute to plasmid yield improvement are reduced metabolic burden during plasmid synthesis; reduced plasmid mediated protein production and altered DNA compaction during plasmid induction. In this article, concise presentations on the optimisation of medium specifically for pcDNA3F cultivation and the development of fed-batch fermentation by strategic glycerol feeding to improve pcDNA3F volumetric and specific yields were presented. In addition, two simple methods to boost plasmid yield based on the alteration of growth pH and temperature fluctuation were also included. A method for pilot-scale fermentation of *E. coli *carrying plasmid DNA measles vaccine construct was successfully developed based on controlled fed-batch fermentation and optimised growth conditions. This method resulted in an improved plasmid based (pcDNA3) measles (F) vaccine volumetric yield of 110 mg/L and specific yield of 14 mg/g. *In vitro *study also showed that the MV-F protein was successfully expressed (data not shown) from pcDNA3F produced using the optimised fed-batch cultivation method.

## Materials and methods

### Materials

Maximum efficiency *E. coli *DH5*α *and pcDNA3 vector were purchased from Invitrogen, VIC, Australia. Tryptone, yeast extract, KH_2_PO_4 _(99.5%) and glucose (99%) were purchased from Merck, NJ, USA. NH_4_Cl (99.5%), ethidium bromide (1%), NaOH (99%), HCl (36.5%), and MgSO_4 _(99.5%) were purchased from Sigma-Aldrich, NSW, Australia. Glycerol (99.5%) and NaCl (99.5%) were purchased from Amresco, OH, USA and disodium hydrogen orthophosphate, Na_2_HPO_4 _(99%) was purchased from Univar, WA, USA.

### Plasmid design

To construct pcDNA3F vaccine, 1.65 kb open reading frame of the measles virus fusion (MV-F) gene from plasmid pTM1-F was amplified by PCR using the oligonucleotides: 5'ACGTAAGC TTACCATGGGTCTCAAGGTGAACGTC 3' and 5' ATGCTCTAGAGCTCAGAGCGACCT TACATAGG 3' containing *Hind*III and *Xba*I restriction enzyme sites respectively. This was followed by ligation into the pcDNA3 vector, and transformation into competent *E. coli *DH5*α *cells.

### Cell propagation and monitoring

10 μL of transformed cells (*E. coli *DH5*α*-pcDNA3F) was cultured in LB-agar-ampicillin plate at 37°C overnight. A single *E. coli *DH5*α*-pcDNA3F colony was picked from the LB-agar-ampicillin plate and subcultured with 250 mL of LB media containing 100 μg/mL ampicillin at 37°C overnight under 200 rpm shaking. Subsequently, 2 mL of the culture was inoculated into 250 mL medium containing 100 μg/mL ampicillin. The batch fermentation was run at 37°C under 200 rpm shaking and was halted at late exponential phase at 15 h. 15 h was selected as the harvest time for batch fermentation as it provided a good combination between optimum cell growth and plasmid production. 1 mL of culture was sampled after every hour for cell growth and plasmid yield analysis.

### Growth media optimisation

2 mL of fresh innoculums were cultured in 500 mL shake flasks containing 250 mL of each of the designated medium (Table 2) at pH 7.0. The culture was incubated at 37°C under 200 rpm shaking, harvested after 15 h and was analysed for plasmid content. The modified PDM medium was known as PDM-pcDNA3F medium. The pcDNA3F volumetric yields were incorporated to multiple non-linear regression analysis to determine the model coefficients. The significance of the models was determined to check their efficiency. All statistical analyses were done using the software Statistica (Statsoft, v. 5.0). The optimum values for tryptone, glucose and Na_2_HPO_4 _were predicted using the steepest ascent method of RSM.

### Effect of pH and temperature on plasmid production and cell growth

All experiments were carried out using the same master cell bank to minimize errors due to cell age. 2 mL fresh inoculums were dispensed into each 500 mL conical flask containing 100 μg/ml ampicillin and 250 mL PDM. The cultures were incubated at 37°C and 200 rpm for 15 h. Effect of pH on plasmid production and cell growth was investigated by varying the initial cultivation pH values between 6 and 8.5. To explore the effect of temperature shift on plasmid production, one step and periodic temperature inductions at 35 and 45°C were performed at 8 h post innoculation at 37°C. For periodic temperature induction, a simple sinusoidal wave model (equation (2)) was used to represent the temperature fluctuation. More specifically, the objective of the temperature fluctuation was to find the frequency *f *at which the plasmid production was optimum. All samples were analysed for plasmid yield and biomass growth periodically.

### Fed-batch fermentation

A single bacterial colony carrying the plasmid pcDNA3F (*E. coli *DH5*α*-pcDNA3F) was picked from an LB-agar-ampicillin plate and subcultured overnight in 500 mL shake flask containing 250 mL PDM-pcDNA3F medium, 1% ampicillin and 0.25% v/v glycerol at 37°C and 200 rpm shaking. Subsequently, the culture was inoculated into a 20 L fermentor (New Brunswick Scientific, BioFlo 410, USA) containing 15 L of PDM-pcDNA3F medium and 0.25% v/v glycerol. The initial temperature was set at 37°C and the DO value which was subjected to a previous optimisation was maintained at 10% by the PID controller. The pH was maintained at 7.5 using 4.0 M NaOH and 1.0 M HCl. The inflowing air was set at 20 psi and foaming was controlled by using polypropylene glycol as antifoam. In the fed-batch phase, a carbon substrate feed containing 50% glycerol was added into the fermentor according to equation (3). Temperature shift from 37 to 45°C was performed at 20 h post culture to further boost the plasmid yield prior to harvesting the culture and finally the fermentation was terminated at 24 h post innoculation. The culture broth was harvested, concentrated by ultrafiltration, packaged and stored at -75°C.

### Plasmid DNA analysis

Pure plasmid DNA was prepared using Wizard Plus SV Minipreps according to the manufacturer's instruction (Promega, U.S.A). Clarified cell lysate containing plasmid DNA was prepared according to the protocol described in the Wizard Plus SV Minipreps manual. Briefly, 1 mL of cell culture was resuspended, alkaline lysed, neutralised and clarified by centrifugation. The pDNA concentration was determined by ethidium bromide agarose gel electrophoresis using 1 kbp DNA ladder (Bio Labs, New England). The gel was made up in 50-fold diluted TAE buffer (242 g of Tris base, 57.1 mL acetic acid, 9.305 g of EDTA) and stained with 3 μg/mL ethidium bromide. The gel well was loaded with 1 μL of sample and electrophorased at 65 V for 70 min. Consequently, the gel was photographed using a gel analyser (BIORAD, Universal Hood II, Italy) and analysed using Quantity One software (BIORAD, USA).

## List of abbreviations

*a*: parameter's coefficient; *D*: Dilution rate (h^-1^); *f *: Frequency of oscillation (h^-1^); *F_i_*: Initial substrate feeding rate (mL/h); *F_t_*: Substrate feeding rate at time *t *(mL/h); *S_i_*: Initial substrate consumption rate (mL/h); *S_t_*: Substrate consumption rate at time *t *(mL/h); *t*: Cultivation time (h); *t_d_*: Biomass doubling time (h); *T*(*t*): Temperature at time *t *(°C); *x*: Coded value of nutrient concentration; *X*: Real value of nutrient concentration (g/L); *y*: Product concentration (mg/L); *α*: Specific exponential feeding rate (h^-1^); *μ*: Specific cell growth rate (h^-1^); Δ*T*: Temperature's maximum deviation (°C); *π*: pi (3.142); *ϕ*: Phase during oscillation.

## Competing interests

The authors declare that they have no competing interests.

## Authors' contributions

CMO: All experiments and manuscript preparation. RP and DW: Construction of plasmid pcDNA3F. MKD: Research design and manuscript preparation.

All authors have read and approved the final manuscript.
